# Knowledge and Attitudes Are Related to Selected Salt-Specific Behaviours among Australian Parents

**DOI:** 10.3390/nu10060720

**Published:** 2018-06-04

**Authors:** Durreajam Khokhar, Caryl Anne Nowson, Claire Margerison, Bruce Bolam, Carley Ann Grimes

**Affiliations:** 1 Institute for Physical Activity and Nutrition, Deakin University, Locked Bag 20000, Waurn Ponds, Geelong, VIC 3220, Australia; dkhok@deakin.edu.au (D.K.); caryl.nowson@deakin.edu.au (C.A.N.); claire.margerison@deakin.edu.au (C.M.); 2 Department of Health and Human Services, 50 Lonsdale Street, Melbourne, VIC 3000, Australia; bruce.bolam@dhhs.vic.gov.au

**Keywords:** salt, knowledge, attitude, behaviour, parent, Australia

## Abstract

Salt intake in adults and children exceeds recommended levels. Salt-related knowledge, attitudes, and behaviours (KABs) may influence the amount of salt consumed. The aims of this study were to assess salt-related KABs among parents, and investigate whether salt-related knowledge and attitudes are associated with salt-specific behaviours. Parents with children <18 years were recruited from four shopping centers across Victoria, Australia; Facebook; and an online consumer research panel; they then completed an online questionnaire assessing salt-related KABs and salt use in children. Eight hundred and thirty-seven parents (mean age 41.0 (10.0) (SD) years) provided valid responses. Most (77%) parents were aware that eating too much salt damages children’s health and that reducing the amount of salt in their children’s diet was important (70%), and 46% reported adding salt to food prepared for their children. Parents who were aware that eating too much salt damages children’s health were less likely to report that their child added salt at the table (OR = 0.51, *p* < 0.001), and that they added salt to food prepared for the child (OR = 0.46, *p* < 0.001). Educational messages that highlight the adverse health effects of salt during childhood are likely to be useful in reducing discretionary salt use in the home environment.

## 1. Introduction

Currently, dietary salt intake among Australian adults is between 8–9 g/day [1], which is well in excess of the 5 g/day recommended by the World Health Organization (WHO) [2]. Similarly, Australian primary schoolchildren aged 4–12 years are also consuming high levels of salt, with mean intakes of 6 g/day [3]. There is conclusive evidence that excess salt intake is associated with raised blood pressure in adults [4,5,6], and is a major risk factor for the development of cardiovascular disease (CVD) [7]. Whilst the prevalence of CVD-related conditions is greater among middle-aged adults, certain risk factors, such as high salt intakes from a young age, may contribute to the development of premature CVD-related conditions [2].

In 2013, the Federal Government of Australia signed up to the WHO’s global target to reduce population salt intake by 30% by 2025 [8]. To ensure that Australia accomplishes the global target set out by the WHO, the implementation of a multi-faceted salt reduction strategy is warranted. In 2015, the Victorian Health Promotion Foundation (VicHealth) and the Heart Foundation launched an initiative to reduce population-wide salt intakes in the state of Victoria [9]. The key components of this initiative include engagement with the food industry, as well as consumer education, research, and social marketing strategies [9]. Previous evidence from the United Kingdom’s (UK) salt reduction strategy suggests that understanding the impact that knowledge and attitudes have on behaviour is important, as this can be used to inform strategies that may lead to a reduction in population salt intake [10]. Whilst a number of studies worldwide have assessed salt-related knowledge, attitudes and behaviours (KABs) in adults [11,12,13,14,15,16,17,18,19,20,21,22,23], studies assessing KABs specifically in parents are limited [24]. The importance of this data cannot be underestimated, because parents have a major role in influencing children’s eating behaviours [25,26]. Therefore, understanding parents’ salt-related knowledge and attitudes and their influence on behaviour can help to inform the planning, design, and implementation of future consumer salt reduction initiatives in Australia, including current initiatives underway in the state of Victoria. Thus, the objectives of this study were to assess salt-related KABs among parents of children under 18 years of age, and to investigate whether their salt-related knowledge or attitudes were associated with the salt-specific behaviours of both parents and children. 

## 2. Materials and Methods

### 2.1. Study Design and Recruitment

This was a cross-sectional study completed in a sample of Victorian residents aged 18–65 years. Full details of the methodology have been described elsewhere [14]. In brief, recruitment was conducted from September to November 2015 with participants recruited using three strategies: an intercept survey within four shopping centers across Victoria, a Facebook advertisement campaign, and an online consumer research panel (Lightspeed GMI). Consent was obtained by all of the participants before participation in the study. Ethics approval for this study was granted by the Deakin University Human Ethics Advisory Group (Project No. HEAG-H 83_2015).

Participants were asked to complete an online questionnaire assessing salt-related KABs. Qualtrics (Provo, UT, USA), an online survey software instrument, was used to deliver the questionnaire. Within the questionnaire, participants were asked to indicate if they were parents or caregivers of a child or children <18 years of age. Those that responded “yes” were included in the present study; details on the full sample have previously been reported [14].

### 2.2. Shopping Center Recruitment and Data Collection

To capture a representative spread of participants across different socio-economic status (SES) stratum, participants were recruited from shopping centers from geographic areas of varying socio-economic advantage and disadvantage, as indicated by the Socio-Economic Indexes for Areas (SEIFA) indices [27]. One shopping center site was recruited from the bottom and two were recruited from the top tertile in Greater Melbourne; and one site was recruited from the bottom tertile in Geelong. The postcode of each shopping center was matched with the corresponding “Index of Relative Socio-Economic Advantage and Disadvantage” (IRSAD) at the state level for Victoria [27] to group into tertiles of SEIFA. The survey was conducted over 26 days, including each day of the week, between September–November 2015. The researchers approached passing shoppers and invited them to participate in the study. In the case where shoppers declined to participate, the researcher would approach the next passing shopper.

### 2.3. Facebook Advertisement Campaign

An eight-week Facebook advertisement campaign was launched as a recruitment strategy from mid-September to mid-November 2015. Advertisements were displayed to Facebook users who were Victorian residents aged 18–64 years. The age group options on Facebook are limited to 18–64 years or 18–65+ years. Thus, the former age range was selected for the present study. Clicking on the advertisement redirected the Facebook user to the Qualtrics webpage to complete the survey.

### 2.4. GMI Consumer Research Panel

Participants were recruited via an online panel provider, Lightspeed GMI. The GMI research is a consumer panel of individuals who have voluntarily registered themselves to participate in market research studies and earn reward points that they can redeem for monetary gifts and donations. Participants were emailed a letter containing a URL link to the Plain Language Statement and consent form. Participants were then able to access the questionnaire via the Qualtrics link provided.

### 2.5. Survey Instrument

A questionnaire containing 37 questions was developed to assess KABs related to dietary salt intake (Supplementary File 1). These questions were modelled on those used in previous salt-related surveys [11,15,21,28,29,30,31,32,33,34]. Eleven questions assessed demographic characteristics, 18 questions assessed overall KABs related to dietary salt intake, and eight questions specifically assessed KABs related to children’s salt intake. We have previously reported findings from the 18 questions that assessed salt-related KABs more broadly among adults [14]. The main focus of this study pertains to the eight additional questions answered by the participants who identified themselves as parents/caregivers with children <18 years. In addition to reporting on these eight questions (described below), we present the findings from eight out of the 18 questions that assessed salt-related KABs more broadly among adults. Six out of these eight questions were selected to assess whether salt-related knowledge (two questions) was associated with salt-specific behaviours (four questions). These questions were selected based on the rationale that previous studies suggested that certain knowledge is favorably associated with certain salt-related behaviours [15,20]. The other two attitude questions were included, as these are popular questions included in previous KABs studies that aid in the understanding of parents’ perception of their own salt intakes as well as the level of concern regarding salt compared to other nutrients such as fat, sugar, etc.

#### 2.5.1. Demographic Characteristics

The demographic characteristics section of the survey comprised of questions related to parents’ age, gender, level of education, country of birth, languages spoken, history of chronic disease, and self-reported height and weight. Body mass index (BMI) was used to categorize participants into weight categories (e.g., underweight (BMI < 18.5 kg/m^2^), healthy weight (BMI 18.5–24.9 kg/m^2^), overweight (BMI 25.0–29.9 kg/m^2^), and obese (BMI ≥ 30.0 kg/m^2^)) [35]. Socio-economic status (SES) was based on educational attainment, and was defined as (1) low SES: those with some or no level of high school education; (2) mid SES: those with a technical/trade certificate or diploma; and (3) high SES: those with a university/tertiary qualification. One additional demographic question asked parents about the age range of children in their care: 0–1 year, 2–4 years, 5–12 years, and 13–17 years. In the case of the parent having more than one child aged <18 years in their care, more than one age category could be selected. To describe these characteristics, parents were grouped based on the age of children in their care. Parents that reported having a child/children in either the 0–1 and/or 2–4 years age group were categorized into the ‘Infants/toddlers/preschoolers’ age group, while those that had reported having a child/children in the 5–12 and/or 13–17 years age group were categorized into the ‘Mid-childhood/adolescence’ age group. Those parents who had a child/children in both of these age groups were categorized into the ‘Both’ age group.

#### 2.5.2. Knowledge Related to Salt Intake

##### Parents’ Knowledge Related to Children’s Salt Intake

Two questions addressed parents’ knowledge related to their children’s salt intakes, both of which used a five-point Likert scale. One question related to their awareness of salt intake among Australian children (In general, how much salt do you think Australian children eat?) with response options ranging from ‘far too much’ to ‘far too little’ or ‘don’t know’. The second knowledge question related to parent’s awareness of whether long term excess salt intakes damage children’s health (In the long term, eating too much salt during childhood may have harmful effects on children’s health) with response options ranging from ‘strongly disagree’ to ‘strongly agree’.

##### Parents’ Knowledge Related to the Salt Intake of Adults

One question assessed parent’s knowledge related to their awareness of salt intake among Australians more generally (In general, how much salt do you think Australians eat?) with response options ranging from ‘far too much’ to ‘far too little’ or ‘don’t know’. The second knowledge question related to awareness of whether excess salt intake could damage health (Do you think that eating too much salt could damage your health?). Response options included ‘yes’, ‘no’, and ‘don’t know’. For analysis, the response option ‘don’t know’ was combined with ‘no’, hence creating a dichotomous variable of either ‘yes’, which indicated a correct response or ‘no/don’t know’, which indicated an incorrect response.

For the other knowledge questions with response options ‘far too much’, ‘too much’, ‘just the right amount’, ‘too little’, ‘far too little’, or ‘don’t know’, the responses ‘far too much/too much’ and ‘too little/far too little’ were collapsed into one category for the purpose of descriptive analysis. For bivariate analyses that assessed the association between knowledge and salt-specific behaviours, the responses were collapsed into a dichotomous variable i.e., ‘far too much/‘too much’ were grouped together and indicated correct knowledge, whereas ‘too little/far too little/just the right amount/don’t know’ were grouped together and indicated incorrect knowledge.

#### 2.5.3. Attitudes Related to Salt Intake

##### Parents’ Attitudes Related to Children’s Salt Intake

Two questions addressed the attitudes of parents related to children’s salt intake, both of which used a five-point Likert scale for the following statement and question: ‘Limiting the amount of salt my child or children eat/s is important to me’ and ‘Do you think more action needs to be taken to reduce the salt in foods targeted at children?’, with response options ranging from ‘strongly disagree’ to ‘strongly agree’.

##### Parents’ Attitudes Related to Salt intake of Adults

Two questions addressed the attitudes of parents related to salt intake in adults. The first question asked about the perception of parents’ own salt intakes (‘How do you think your daily salt intake compares to the amount of salt recommended by health professionals?). Response options included ‘I eat less salt than recommended’, ‘I eat about the right amount of salt’, ‘I eat more salt than recommended’, or ‘I don’t know’. The second question asked parents to indicate their level of concern regarding six food-related issues: healthy eating, the amount of sugar in food, the amount of salt in food, the amount of fat in food, the amount of saturated fat in food, and the amount of kilojoules/calories in food. A five-point Likert scale was used, with response options ranging from ‘not at all concerned’ to ‘extremely concerned’. For descriptive analysis, the responses ‘not at all concerned/not very concerned’ and ‘very concerned/extremely concerned’ were collapsed into one category.

For those questions where response options were ‘strongly disagree’, ‘disagree’, ‘neither agree nor disagree’, ‘agree’, or ‘strongly agree’, the responses ‘strongly disagree/disagree’ and ‘strongly agree/agree’ were collapsed into one category for the purpose of descriptive analysis. For bivariate analyses, the response option ‘neither agree nor disagree’ was combined with ‘strongly disagree/disagree’, hence creating a dichotomous variable of either ‘strongly agree/agree’, which indicated a correct response/positive attitude, or ‘strongly disagree/disagree/neither agree nor disagree’, which indicated an incorrect response/negative attitude.

#### 2.5.4. Behaviours Related to Salt Intake

##### Salt-Related Behaviours of Parents Specific to Their Children

Three questions addressed parents’ behaviours related to the discretionary salt use of their child/children, which included the frequency of adding salt to food prepared for their child/children, placing a salt shaker on the table at meal time, and the frequency of the child/children adding salt at the table (Supplementary File 1). A five-point Likert scale was used, with response options ranging from ‘always’ to ‘never’.

##### Parents’ Own Salt-Related Behaviours

Three questions assessed behaviours related to the parent’s own discretionary salt use (adding salt during cooking, adding salt at the table (not specified for food prepared for children or child adding salt at the table), and placing a salt shaker on the table at meal times). A five-point Likert scale was used with response options ranging from ‘always’ to ‘never’.

An additional behaviour question asked parents to report the frequency of engaging in various behaviours related to reducing salt in their diet over the past month. These behaviours included looking at a food label to check the salt/sodium content on a food label, purchased foods labelled ‘no added salt’, ‘salt reduced’, or ‘reduced sodium’, avoided eating packaged, ready-to-eat, foods, avoided eating from fast food restaurants or takeaway stores, used herbs and spices instead of salt when cooking, and asked for a meal to be prepared without salt when eating out. Response options ranged from ‘always do this’ to ‘never do this’, and ‘does not apply to me’ was also an option.

For questions with response options ‘always’, ‘often’, ‘sometimes’, ‘rarely’, and ‘never’, the responses ‘always/often’ and ‘rarely/never’ were collapsed into one category. For bivariate analyses that assessed the association between knowledge or attitudes and salt-specific behaviours, the response option ‘sometimes’ was combined with the ‘always/often’ response, grouping responses as either ‘always/often/sometimes’ and ‘rarely/never’. The response ‘does not apply to me’ was excluded from analysis due to a low number of parents selecting this option.

### 2.6. Data Analysis

All of the data obtained from the questionnaires were analyzed using Stata/SE (StataCorp LP) version 14.0 (TX, USA). Descriptive statistics were performed for demographic variables. Continuous and categorical data were expressed as mean (±standard deviation (SD)) or number of participants (*n*) and percentages, respectively. Chi-square analyses were performed to determine whether knowledge or attitudes were associated with behaviours. To adjust for potential confounders of parent’s age, sex and SES logistic regression models, with corresponding odds ratios (OR) and 95% confidence intervals (CI), were also performed. Four knowledge questions (two related to parents’ knowledge about salt in the diet overall, and two related to knowledge about salt in children’s diets) as well as one attitude question (related to importance of limiting salt in children’s diets) were selected to examine their association with discretionary salt use (parent and child) and other salt reduction related behaviours (e.g., checking salt content on food labels). These questions were selected as previous studies have shown that certain salt-related knowledge and attitudes are associated with salt-specific behaviours [15,20].

## 3. Results

### 3.1. Demographic Characteristics

A total of 840 parents/caregivers, with at least one child <18 years, completed the online survey. Three parents were excluded as they had not completed any of the additional parent questions, resulting in a total of 837 parents with complete survey responses. Over half (59%) of the parents were females, and the mean age of the sample was 41(10) years (SD) (Table 1). The majority of parents were born in Australia and spoke English as their primary language.

### 3.2. Knowledge

**Figure 1:**
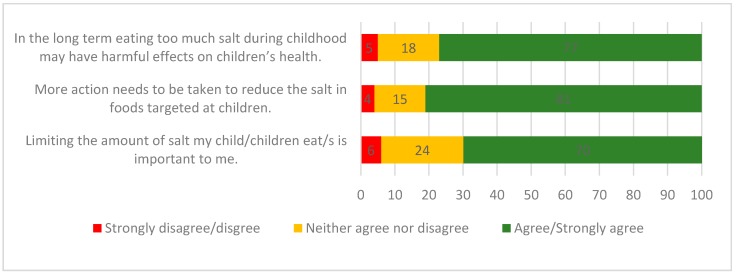
Parents’ knowledge and attitudes towards salt intake related to their child/children (*n* = 837).

Overall, 84% of parents were aware that Australians eat too much salt, whereas fewer (73%) parents were aware that Australian children eat too much salt. Similarly, most parents (91%) were aware that eating too much salt could damage health, whilst fewer (77%) agreed that in the long term, excess salt intake during childhood may be harmful to children’s health (Figure 1).

### 3.3. Attitudes

Just over half (66%) of the parents believed that their own salt intake was either equal to or below the recommended daily level, and approximately one third (34%) believed their own salt intake exceeded the recommendations. About half of the parents (49%) were very/extremely concerned about the amount of salt in their diet. More parents reported that they were very/extremely concerned about the amount of sugar (63%), amount of saturated fat (62%), healthy eating (56%), and amount of fat (55%) in their diet, whereas fewer parents (42%) were concerned about the amount of kilojoules in their diet. With regards to concern about salt in their child’s diet, more than two thirds (70%) of parents agreed that limiting the amount of salt that their child ate was important to them and the majority (81%) agreed that more action needs to be taken to reduce the amount of salt targeted at children (Figure 1).

### 3.4. Behaviours

**Figure 2:**
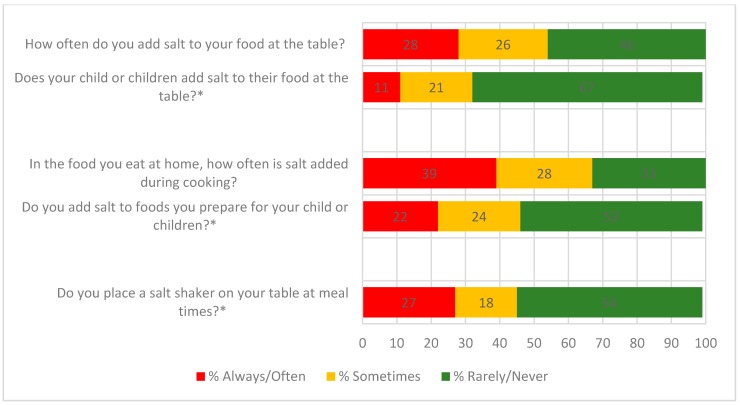
Discretionary salt use behaviours of parents and those related to their child/children. * Parent reported behaviour for child/children in the parent-specific component of the questionnaire.

About half of all parents (54%) reported that they added salt to their own food at the table, while about one third (32%) reported that their child/children added salt to food at the table (Figure 2). Just over two-thirds of parents (67%) reported that they added salt during cooking, whereas comparatively fewer (46%) reported that salt was added to foods prepared for their child/children. Forty five percent of parents reported placing a salt shaker on the table at meal times.

With regards to actions taken to reduce salt in their diet in the last one month, the most commonly reported behaviours included always or often ‘using spices and herbs as an alternative to salt in cooking’ (53%), ‘avoided eating from fast food restaurants’ (50%), and ‘avoided eating packaged, ready-to-eat foods’ (46%). Fewer parents reported that they always or often ‘purchased foods labelled no added or reduced salt/sodium’ (39%), ‘avoided eating from Asian-style restaurants or takeaway stores’ (33%), ‘looked at a food label to check the sodium/salt content of a food’ (31%), or ‘asked to have meal prepared without salt when eating out’ (16%) (Table S1).

#### Association of Salt-Related Knowledge and Attitudes with Behaviours

**Figure 3:**
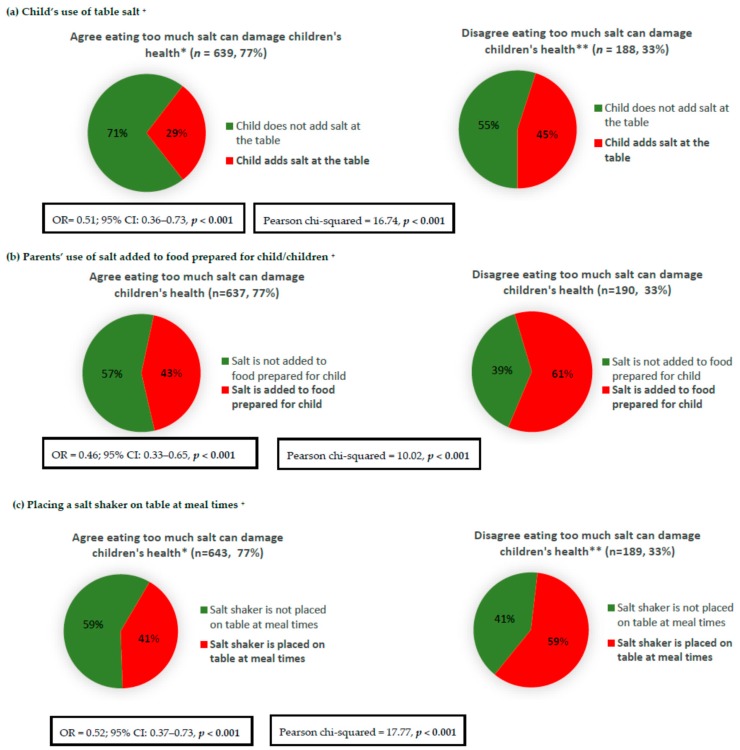
Association between parents’ knowledge of the long term health implications of eating too much salt during childhood and discretionary salt use behaviours related to their child/children. * Figure 3 responses include ‘strongly agree’ and ‘agree’; ** Figure 3 responses include ‘strongly disagree’, ‘disagree’, and ‘neither agree nor disagree’; ^+^ Logistic regression adjusted for parent’s age, sex, and SES.

Parents who agreed that in the long term, eating too much salt during childhood may have harmful effects on children’s health, were less likely to report discretionary salt use behaviours for their children, e.g., less likely for the child to add salt at the table, less likely to add salt to food prepared for the child, and less likely to place a salt shaker on the table at meal times (Figure 3). After adjustment for confounding factors, including parent’s age, sex, and SES, these associations remained significant (Figure 3). For example, parents who agreed that harm could result from children eating too much salt were 51% less likely to report that their child adds salt at the table (OR = 0.51; 95% CI: 0.36–0.73). Furthermore, in adjusted analyses (Table 2), these parents were more likely to report engaging favorably in five out of the seven salt reduction related behaviours examined (e.g., avoided eating packaged, ready-to-eat foods; avoided eating from fast food restaurants; looked at food label to check sodium/salt content; purchased foods labelled as ‘no added or reduced salt/sodium’; and used spices and herbs instead of salt in cooking), but were less likely to ask for their meal to be prepared without salt when eating out. No association was found between the possession of this knowledge and avoiding eating from Asian-style restaurants (*p* = 0.119) Associations between parents’ knowledge that was specific to salt intake in adults and their own discretionary salt use behaviours as well as other salt reduction related behaviours after adjustment for confounding factors are reported in Table 3. Overall, the same pattern was observed, whereby parents who were aware that eating too much salt could damage health were less likely to report discretionary salt use and more likely to report engaging favorably in a range of salt reduction related behaviours (Table 3).

**Figure 4:**
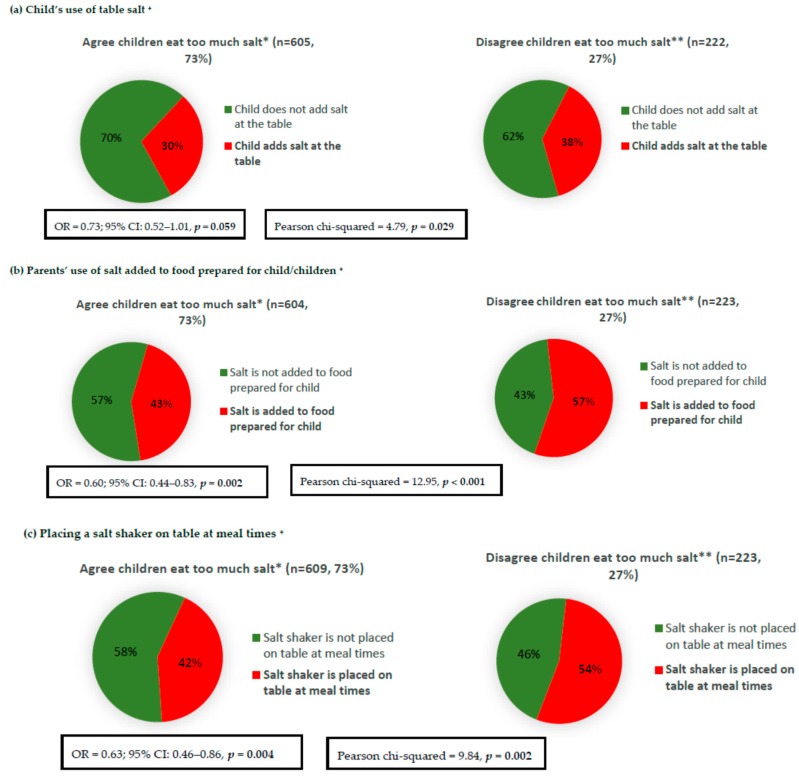
Association between parents’ knowledge that Australian children eat too much salt and discretionary salt use behaviours related to their child/children. * Figure 4 responses include ‘far too much’ and ‘too much’; ** Figure 4 responses include ‘just the right amount’, ‘too little’, ‘far too little’, and ‘don’t know’; ^+^ Logistic regression adjusted for parent’s age, sex, and SES.

Parents who were aware that Australian children eat too much salt were less likely to report discretionary salt use for their children, e.g., the child’s use of salt at table, adding salt to food prepared for the child, and placing a salt shaker on the table at meal times (Figure 4). The associations remained significant (Figure 4) after adjustment for parent’s age, sex, and SES, with the exception of the child’s salt use at the table (OR = 0.73; 95% CI: 0.52–1.01, *p* = 0.059). Furthermore, in adjusted analyses, knowledgeable parents were more likely to engage in only one out of the seven examined salt reduction related behaviours; parents who were aware that Australian children eat too much salt were 1.5 times more likely to buy foods labelled ‘no added or reduce salt/sodium’ (OR = 1.55; 95% CI: 1.11–2.16) (Table 2). With regards to parent’s knowledge specific to salt intake in adults, overall, the same pattern was observed in the adjusted analysis, whereby parents who were aware that Australians eat too much salt were less likely to use discretionary salt and engage favorably in one out of the seven salt reduction related behaviours (Table 3). Unadjusted analysis of the associations between parents’ knowledge specific to the salt intake of adults and their own discretionary salt use behaviours as well as other salt reduction related behaviours are reported in Table S2.

**Figure 5:**
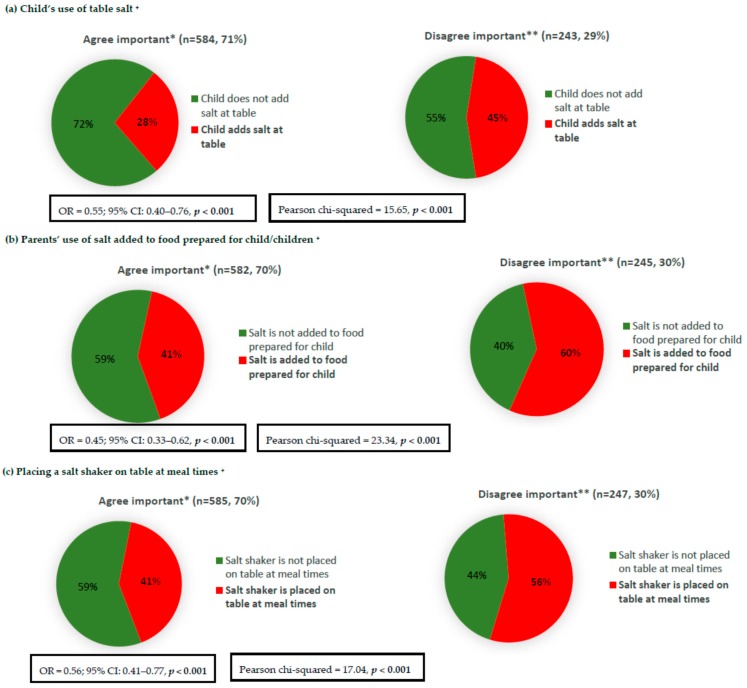
Association between parents’ attitude towards the importance of limiting the amount of salt their child eats and discretionary salt use behaviours related to their child/children. * Figure 5 responses include ‘strongly agree’ and ‘agree’; ** Figure 5 responses include ‘strongly disagree’, ‘disagree’ and ‘neither agree nor disagree’; ^+^ Logistic regression adjusted for parent’s age, sex, and SES.

Parents who reported that limiting the amount of salt their child eats is important were less likely to report discretionary salt use in children, e.g., the child’s use of salt at table, adding salt to food prepared for the child, and placing a salt shaker on the table at meal times (Figure 5). The associations remained significant (Figure 5) after adjustments for the parent’s age, sex, and SES; for example, parents with a positive attitude (strongly agree/agree) were 45% less likely to add salt to food prepared for their child (OR = 0.45; 95% CI: 0.33–0.62). Parents who considered limiting the amount of salt that their child eats to be important were also more likely to engage in six out of the seven examined salt reduction related behaviours, e.g., avoided eating packaged, ready-to-eat foods, avoided eating from fast food restaurants, looked at a food label to check the sodium/salt content, purchased foods labelled ‘no added or reduced salt/sodium’, avoided eating from Asian-style restaurants, and used spices and herbs as an alternative to salt in cooking (Table 2). Unadjusted analysis of the associations between parents’ knowledge and attitudes related to their child/children with specific salt-related behaviours are presented in Table S3. 

## 4. Discussion

This is the first Australian study among parents to assess associations between salt-related knowledge and attitudes with behaviours. Overall, knowledge related to excess salt intake and adverse health was relatively high among parents, with the majority (91%) aware that excess salt intakes are detrimental to health. This is similar to other studies conducted in adults in Australia (95% agreed) [17], Canada (93% agreed) [13], and Greece (93% agreed) [19]. However, fewer parents (77%) were aware that in the long term, eating too much salt during childhood may have harmful effects on children’s health. This finding is similar to that previously reported by the Australian Division on World Action on Salt and Health (AWASH) in 2008, which assessed salt-related KABs among Australian parents of children aged 0–18 years, and found that 86% of parents were aware that salt has harmful effects on children’s health [24]. Our findings suggest that the message of salt and health in general has reached more parents than the message of salt being detrimental for children’s health. Similarly, the majority of parents (84%) were aware that Australians in general eat too much salt, while fewer (73%) were aware that children eat too much salt.

Our results indicate that the proportion of parents who consider salt reduction to be of importance for their child/children is greater (77%) than the proportion concerned about salt in their own diet (49%). In the previous AWASH study, fewer (53%) parents reported that they were concerned about salt/sodium in children’s diet. However, findings from our study, as well as those by AWASH, indicate that there is strong support from Australian parents (>80% of parents agree), and that more action should be taken to reduce the amount of salt in foods targeted at children [24]. This indicates that parents are likely to support strategies that target salt reduction in children.

A lower proportion of parents have reported salt use by the child at the table, and salt used in food prepared for their child/children in comparison to discretionary salt use for themselves. Approximately two-thirds (67%) of parents reported using salt during cooking; however, fewer (46%) reported using salt during food preparation for children. Similarly, whilst just over half (54%) of parents reported adding salt at the table, comparatively fewer (32%) reported the child’s use of salt at the table. These findings are similar to Sarmugam et al., who assessed discretionary salt use among Australians adults (*n =* 530) [36] and found that 64% and 52% always/usually/sometimes added salt during cooking and at the table, respectively [36]. Similarly, a large cross-sectional study of Victorian primary schoolchildren, the Salt and Other Nutrients in Children (SONIC) study, which examined sodium and potassium urinary excretion and discretionary salt use, found that 28% usually/sometimes reported that the child adds salt to food at the table, while 66% of parents reported usually/sometimes adding salt to food during cooking [3]. Similar to our findings, the SONIC study also found that approximately one-third of parents reported usually/sometimes placing a salt shaker on the table at meal times [3].

We found that salt-related knowledge and attitudes were significantly associated with certain salt-specific behaviours. In particular, parents who were aware of the long term health implications of eating too much salt during childhood were more likely to engage in a number of favorable salt-related behaviours, such as their child not using table salt, not adding salt to food prepared for their children, not placing a salt shaker on the table, buying no added or reduced salt/sodium foods, checking food labels for salt/sodium content, avoiding packaged, ready-to-eat foods, and eating from fast food restaurants.

Whilst parents who were aware that both Australians and children eat too much salt were less likely to report discretionary salt use and more likely to purchase low salt/sodium foods, no significant associations were found with the other salt-related behaviours. Thus, it appears that in order to influence salt reduction related behaviours, it is important that messages regarding the link between high salt intakes and health, specifically in children, are disseminated to parents. These messages may be more important than the awareness that Australian children eat too much salt. Although we found that salt-related knowledge was associated with a number of favorable salt reduction related behaviours—for example parents who were aware of the long term health link with excess salt during childhood were less likely (43%) to add salt to food prepared for children than those parents who did not possess this knowledge (61%)—there still was a large proportion of parents who knew the negative effects of salt on health, and yet were still adding salt to food prepared for their child. This suggests that knowledge may not be the only factor involved in determining behavioral change. The association between knowledge and its impact on behaviour is complex and is influenced by various demographic and environmental factors [37]. Cultural, educational, and economic factors [38,39], as well as generic skills in cooking and food preparation [37], food availability [40], and taste preference [41] have all been shown to influence food choice and consumption [38,42].

Although we found that the majority of parents were aware of the broader link between excess salt intake and health outcomes in adults, we believe that maintaining this existing salt-related knowledge is important, and should still be targeted within future salt reduction campaigns. This is because this knowledge was associated with a number of favorable salt-related behaviours (such as looked at food labels to check sodium content, avoided eating packaged, ready-to-eat foods, used herbs and spices as alternatives to salt during cooking, avoided eating from fast food restaurants, avoided eating from Asian-style restaurants, purchased food labelled ‘no added salt’ or ‘reduced salt/sodium’, and reduced salt use in the home).

With regards to attitudes, parents who agreed that limiting the amount of salt that children eat is important were significantly less likely to report salt use by their child/children at the table, adding salt to food prepared for their child/children, and placing a salt shaker on the table at meal times, and were more likely to engage favorably in almost all of the salt reduction related behaviours (such as looked at food labels to check sodium content, avoided eating packaged, ready-to-eat foods, used herbs and spices as alternatives to salt during cooking, avoided eating from fast food restaurants, avoided eating from Asian-style restaurants, purchased food labelled ‘no added salt’ or ‘reduced salt/sodium’, and reduced salt use in the home). These findings are in line with those reported by Nasreddine et al. in Lebanese adults [20] and Grimes et al. [15] in Australian adults, who also found that participants who were more concerned about salt were more likely to read the salt content on food labels. Thus, improvement in parent attitudes towards salt in the diet may also be an important factor to consider along with knowledge when developing future salt-reduction strategies, as some evidence has previously suggested that increasing nutrition knowledge results in an improvement in attitudes, beliefs, and self-efficacy towards consuming a healthy diet and physical activity [43,44].

### Strengths and Limitations

A strength of this study is the large sample size of 837 parents, who were recruited using three different methods in order to capture a representative spread of participants from varying socio-economic backgrounds. However, there were a number of limitations. This was a cross-sectional study; therefore, no causal inferences can be made. Our study was slightly over-representative of females (59%) and those from a higher socio-economic background (45%); thus, the findings may not be directly generalizable to the Victorian or the Australian population. Furthermore, whilst the study did not utilize a previously validated questionnaire, one of the strengths was that the questions were modelled on previous surveys. Further, the questionnaire collected self-reported KABs. Therefore, it is possible that respondents may be susceptible to social desirability bias, which may influence the participants to respond to questions in a way that they believe will be viewed favorably.

## 5. Conclusions

The findings from this study highlight that a higher proportion of parents were aware of the overall link between salt and health compared with those aware of the long term implications of children eating too much salt. Similarly, a higher proportion of parents were aware that Australian adults eat too much salt, whilst a comparatively lower proportion knew that children eat too much salt. This emphasizes that strategies that target salt reduction among children should be incorporated in future salt reduction campaigns, and our findings indicate that parents are likely to support these strategies, as the majority of parents reported that limiting the amount of salt that their child eats is important, and that more action needs to be taken to reduce the salt in foods targeted at children. Our data highlight that parents’ knowledge and attitudes are associated with various favorable salt-related behaviours. We have identified some key messages (i.e., knowledge of the long term health implications of eating too much salt during childhood and the importance of reducing salt in children’s diet) to be incorporated in the current Victorian statewide consumer awareness campaign being implemented by VicHealth and the Heart Foundation. In addition, our findings can help inform target messages for future salt awareness campaigns. Parents are an important cohort, as their food preferences and eating behaviours provide an opportunity to model good eating habits, and research indicates that children’s eating behaviours are influenced by the family environment. Thus, improving parent’s salt-related knowledge and attitudes may aid in shifting to favorable salt reduction related behaviours and may be a potential means of reducing the currently high salt intake of children.

## Figures and Tables

**Table 1:** Demographic characteristics of parents (*n* = 837).

Characteristic		
	*N* (%)	Mean (±SD)
**Gender**		
Male	347 (41%)	
Female	490 (59%)	
**Age (years)**		41 (10)
**Age group (years)**		
18–24	37 (4%)	
25–34	185 (22%)	
35–44	319 (38%)	
45–54	211 (25%)	
55–66	85 (10%)	
**Country of birth ^1^**		
Australia	686 (83%)	
UK	17 (2%)	
New Zealand	9 (1%)	
Other *	113 (14%)	
**Language spoken other than English ^2^**		
English only	672 (81%)	
Other **	157 (19%)	
**Socioeconomic status ^3^**		
High SES	369 (44%)	
Mid SES	248 (30%)	
Low SES	212 (26%)	
Height (cm)		169 (10)
Weight (kg)		78 (18)
**BMI (kg/m^2^) ^4^**		27 (6)
**Weight Category Distribution** **^4^**		
Underweight	15 (2%)	
Healthy weight	298 (40%)	
Overweight	234 (31%)	
Obese	202 (27%)	
**No. of children in age groups ^5^**		
0–1 years	133	
2–4 years	250	
5–12 years	437	
13–17 years	303	
**Child age group**		
Infant/toddler	196 (23%)	
Mid-childhood/adolescence	505 (60%)	
Both	136 (16%)	

* Includes Italy, Greece, China, Vietnam, Lebanon, and other, please specify; ** Includes Italian, Greek, Cantonese, Mandarin, Arabic, Vietnamese, German, Spanish, and Tagalog; ^1^ *n =* 825 as participants who responded “don’t know” *n =* 2 and “prefer not to answer” *n =* 10 were excluded; ^2^ *n =* 829 as participants who responded “don’t know” *n =* 1 and “prefer not answer” *n =* 7 were excluded; ^3^ *n =* 829 as participants *n =* 8 with missing data were excluded; ^4^ *n =* 749 as participants *n =* 88 with missing data were excluded; ^5^ *n =* 1123 as parents could select more than one age category. SES: socio-economic status; UK: United Kingdom.

**Table 2:** Association of parents’ knowledge and attitudes related to their child/children with specific salt-related behaviours (logistic regression).

Variables	Looked at Food Labels to Check Sodium Content (*Yes*) *	Avoided Eating Packaged, Ready-To-Eat Foods (*Yes*) *	Used Spices and Herbs Instead of Salt When Cooking (*Yes*) *	Avoided Eating from Fast Food Restaurants (*Yes*) *	Avoided Eating from Asian-Style Restaurants or Takeaway Store (*Yes*) *	Purchased Foods Labelled ‘No Added Salt’, ‘Reduced Salt/Sodium’ (*Yes*) *	When Eating out, Asked to Have Meal Prepared without Salt (*Yes*) *
	OR [95% CI]	OR [95% CI]	OR [95% CI]	OR [95% CI]	OR [95% CI]	OR [95% CI]	OR [95% CI]
**In the long term eating too much salt may have harmful effects on children’s health**							
**Disagree ^1^**	1.00 (referent)	1.00 (referent)	1.00 (referent)	1.00 (referent)	1.00 (referent)	1.00 (referent)	1.00 (referent)
**Agree ^2^**	**1.73 (1.23–2.42)**	**1.82 (1.24–2.65)**	**1.66 (1.11–2.48)**	**1.52 (1.03–2.25)**	1.32 (0.93–1.88)	**2.34 (1.65–3.32)**	**0.58 (0.41–0.85)**
**How much salt do you think Australian children eat?**							
**Too little ^3^**	1.00 (referent)	1.00 (referent)	1.00 (referent)	1.00 (referent)	1.00 (referent)	1.00 (referent)	1.00 (referent)
**Too much ^4^**	1.20 (0.87–1.65)	1.11 (0.76–1.61)	1.29 (0.87–1.90)	1.03 (0.70–1.51)	0.95 (0.68–1.34)	**1.55 (1.11–2.16)**	**0.45 (0.32–0.63)**
**Limiting the amount of salt my child eats is important to me**							
**Disagree ^1^**	1.00 (referent)	1.00 (referent)	1.00 (referent)	1.00 (referent)	1.00 (referent)	1.00 (referent)	1.00 (referent)
**Agree ^2^**	**1.88 (1.38–2.57)**	**2.07 (1.46–2.94)**	**1.73 (1.19–2.52)**	**1.82 (1.27–2.60)**	**1.81 (1.31–2.49)**	**2.75 (1.99–3.80)**	0.82 (0.58–1.16)

* Response includes ‘always do this’, ‘often do this’, ‘sometimes do this’; ^1^ Response includes ‘strongly disagree’, ‘disagree’, ‘neither agree nor disagree’; ^2^ Response includes ‘strongly agree’, ‘agree’; ^3^ Response includes ‘far too little’, ‘just the right amount’, ‘too little’, and ‘don’t know’; ^4^ Response includes ‘far too much’, ‘too much’; bolded values represent statistical significance at *p* < 0.05; logistic regression models adjusted for parent’s age, sex, and SES.

**Table 3:** Association of parents’ knowledge with specific salt-related behaviours (logistic regression).

	Adding Salt at the Table (*Yes*) *	Adding Salt during Cooking (*Yes*) *	Placing Salt Shaker on Table at Meal Times (*Yes*) *	Looked at Food Labels to Check Sodium Content (*Yes*) *	Avoided Eating Packaged, Ready-To-Eat Foods (*Yes*) *	Used Spices and Herbs Instead of Salt When Cooking (*Yes*) *	Avoided Eating from Fast Food Restaurants (*Yes*) *	Avoided Eating from Asian-Style Restaurants or Takeaway Store (*Yes*) *	Purchased Foods Labelled ‘No Added Salt’, ‘Reduced Salt/Sodium’ (*Yes*) *	When Eating out, Asked to Have Meal Prepared without Salt (*Yes*) *
	OR [95% CI]	OR [95% CI]	OR [95% CI]	OR [95% CI]	OR [95% CI]	OR [95% CI]	OR [95% CI]	OR [95% CI]	OR [95% CI]	OR [95% CI]
**Do you think eating too much salt could damage your health**										
**No ^1^**	1.00 (referent)	1.00 (referent)	1.00 (referent)	1.00 (referent)	1.00 (referent)	1.00 (referent)	1.00 (referent)	1.00 (referent)	1.00 (referent)	1.00 (referent)
**Yes**	**0.48 (0.36–0.65)**	**0.57 (0.41–0.80)**	**0.52 (0.39–0.70)**	**3.13 (1.84–5.35)**	**3.10 (1.82–5.26)**	**2.30 (1.34–3.96)**	**2.08 (1.21–3.59)**	**1.91 (1.14–3.23)**	**3.20 (1.92–5.32)**	1.02 (0.59–1.78)
**How much salt do you think Australian’s eat?**										
**Too little ^2^**	1.00 (referent)	1.00 (referent)	1.00 (referent)	1.00 (referent)	1.00 (referent)	1.00 (referent)	1.00 (referent)	1.00 (referent)	1.00 (referent)	1.00 (referent)
**Too much ^3^**	**0.60 (0.48–0.76)**	**0.60 (0.47–0.78)**	**0.71 (0.56–0.89)**	0.96 (0.65–1.41)	1.14 (0.72–1.79)	1.19 (0.74–1.91)	1.25 (0.79–1.98)	0.93 (0.62–1.41)	**1.83 (1.23–2.72)**	**0.49 (0.33–0.74)**

* Response includes ‘always do this’, ‘often do this’, ‘sometimes do this’; ^1^ Response also includes ‘don’t know’; ^2^ Response includes ‘far too little’, ‘just the right amount’, ‘too little’, and ‘don’t know’; ^3^ Response includes ‘far too much’, ‘too much’; bolded values represent statistical significance at *p* < 0.05; logistic regression models adjusted for parent’s age, sex, and SES.

## Supplementary Materials

The following are available online at http://www.mdpi.com/2072-6643/10/6/720/s1, Supplementary file 1. Salt-related knowledge, attitudes and behaviours questionnaire; Table S1: Proportion of parents who reported engaging in specific behaviours to reduce salt in their diet over the past month (*n =* 837); Table S2: Association of parents’ knowledge related to salt intake in adults and salt-related behaviours; Table S3: Association of parents’ knowledge and attitudes related to their child/children with specific salt-related behaviours.

## References

[1] Land M.A., Neal B.C., Johnson C., Nowson C.A., Margerison C., Petersen K.S. (2018). Salt consumption by Australian adults: A systematic review and meta-analysis. Med. J. Aust..

[2] World Health Organization (2013). Guideline: Sodium Intake for Adults and Children.

[3] Grimes C.A., Riddell L.J., Campbell K.J., Beckford K., Baxter J.R., He F.J., Nowson C.A. (2017). Dietary intake and sources of sodium and potassium among Australian schoolchildren: Results from the cross-sectional Salt and Other Nutrients in Children (SONIC) study. BMJ Open.

[4] Aburto N.J., Ziolkovska A., Hooper L., Elliott P., Cappuccio F.P., Meerpohl J.J. (2013). Effect of lower sodium intake on health: Systematic review and meta-analyses. BMJ.

[5] He F.J., Li J., Macgregor G.A. (2013). Effect of longer term modest salt reduction on blood pressure: Cochrane systematic review and meta-analysis of randomised trials. BMJ.

[6] He F.J., MacGregor G.A. (2004). Effect of longer-term modest salt reduction on blood pressure. Cochrane Database Syst. Rev..

[7] World Health Organization (2013). A Global Brief on Hypertension: Silent Killer, Global Public Health Crisis.

[8] World Health Organization (2013). Global Action Plan for the Prevention and Control of NCDs 2013–2020.

[9] Victorian Health Promotion Foundation (2015). State of Salt: The Case for Salt Reduction in Victoria.

[10] He F.J., Brinsden H.C., MacGregor G.A. (2014). Salt reduction in the United Kingdom: A successful experiment in public health. J. Hum. Hypertens..

[11] Arcand J., Mendoza J., Qi Y., Henson S., Lou W., L’Abbe M.R. (2013). Results of a national survey examining Canadians’ concern, actions, barriers, and support for dietary sodium reduction interventions. Can. J. Cardiol..

[12] Charlton K., Yeatman H., Houweling F., Guenon S. (2010). Urinary sodium excretion, dietary sources of sodium intake and knowledge and practices around salt use in a group of healthy Australian women. Aust. N. Z. J. Public Health.

[13] Claro R.M., Linders H., Ricardo C.Z., Legetic B., Campbell N.R. (2012). Consumer attitudes, knowledge, and behavior related to salt consumption in sentinel countries of the Americas. Rev. Panam. Salud Publica.

[14] Grimes C., Kelley S.J., Stanley S., Bolam B., Wesbter J., Khokhar D., Nowson C.A. (2017). Knowledge, attitudes and behaviours related to dietary salt among adults in the state of Victoria, Australia 2015. BMC Public Health.

[15] Grimes C.A., Riddell L.J., Nowson C.A. (2009). Consumer knowledge and attitudes to salt intake and labelled salt information. Appetite.

[16] Johnson C., Mohan S., Rogers K., Shivashankar R., Thout S.R., Gupta P., He F.J., MacGregor G.A., Webster J., Krishnan A. (2017). The Association of Knowledge and Behaviours Related to Salt with 24-h Urinary Salt Excretion in a Population from North and South India. Nutrients.

[17] Land M.A., Webster J., Christoforou A., Johnson C., Trevena H., Hodgins F., Chalmers J., Woodward M., Barzi F., Smith W. (2014). The association of knowledge, attitudes and behaviours related to salt with 24-hour urinary sodium excretion. Int. J. Behav. Nutr. Phys. Act..

[18] Magalhaes P., Sanhangala E.J., Dombele I.M., Ulundo H.S., Capingana D.P., Silva A.B. (2015). Knowledge, attitude and behaviour regarding dietary salt intake among medical students in Angola. Cardiovasc. J. Afr..

[19] Marakis G., Tsigarida E., Mila S., Panagiotakos D.B. (2014). Knowledge, attitudes and behaviour of Greek adults towards salt consumption: A Hellenic Food Authority project. Public Health Nutr..

[20] Nasreddine L., Akl C., Al-Shaar L., Almedawar M.M., Isma’eel H. (2014). Consumer knowledge, attitudes and salt-related behavior in the Middle-East: The case of Lebanon. Nutrients.

[21] Papadakis S., Pipe A.L., Moroz I.A., Reid R.D., Blanchard C.M., Cote D.F., Mark A.E. (2010). Knowledge, attitudes and behaviours related to dietary sodium among 35- to 50-year-old Ontario residents. Can. J. Cardiol..

[22] Webster J.L., Li N., Dunford E.K., Nowson C.A., Neal B.C. (2010). Consumer awareness and self-reported behaviours related to salt consumption in Australia. Asia Pac. J. Clin. Nutr..

[23] Zhang J., Xu A.Q., Ma J.X., Shi X.M., Guo X.L., Engelgau M., Yan L.X., Li Y., Li Y.C., Wang H.C. (2013). Dietary sodium intake: Knowledge, attitudes and practices in Shandong Province, China, 2011. PLoS ONE.

[24] Australian Division of World Action on Salt and Health (2008). 2008 Consumer Survey on Parents’ Attitudes to Salt and Children.

[25] Birch L., Savage J.S., Ventura A. (2007). Influences on the Development of Children’s Eating Behaviours: From Infancy to Adolescence. Can. J. Diet Pract. Res..

[26] Savage J.S., Fisher J.O., Birch L.L. (2007). Parental influence on eating behavior: Conception to adolescence. J. Law Med. Ethics.

[27] Australian Bureau of Statistics (2013). Census of Population and Housing: Socio-Economic Indexes for Areas (SEIFA), Australia, 2011.

[28] Australian Division of World Action on Salt and Health (2007). 2007 Survey of Australian Consumer Awareness and Practices Relating to Salt Report.

[29] Consensus Action on Salt & Health (2010). Consensus Action on Salt and Health and Your Health—TNS Public Opinion Survey Summary Report. http://www.actiononsalt.org.uk/Docs/39307.pdf.

[30] Food Standards Agency & COI Comminucations (2005). Consumer Attitudes to Food Standards Wave 5.

[31] New Zealand Food Safety Authority (2011). Salt Consumer Survey. http://www.foodsafety.govt.nz/elibrary/industry/salt-survey.pdf.

[32] Newson R.S., Elmadfa I., Biro G., Cheng Y., Prakash V., Rust P., Barna M., Lion R., Meijer G.W., Neufingerl N. (2013). Barriers for progress in salt reduction in the general population. An international study. Appetite.

[33] Sarmugam R., Worsley A., Flood V. (2014). Development and validation of a salt knowledge questionnaire. Public Health Nutr..

[34] World Health Organisation (2015). WHO Steps Instrument (Core and Expanded) v3. http://www.who.int/chp/steps/instrument/en/.

[35] World Health Organisation (2016). BMI Classification.

[36] Sarmugam R., Worsley A., Wang W. (2013). An examination of the mediating role of salt knowledge and beliefs on the relationship between socio-demographic factors and discretionary salt use: A cross-sectional study. Int. J. Behav. Nutr. Phys. Act..

[37] Parmenter K., Waller J., Wardle J. (2000). Demographic variation in nutrition knowledge in England. Health Educ. Res..

[38] Dallongeville J., Marecaux N., Cottel D., Bingham A., Amouyel P. (2001). Association between nutrition knowledge and nutritional intake in middle-aged men from Northern France. Public Health Nutr..

[39] Wardle J., Parmenter K., Waller J. (2000). Nutrition knowledge and food intake. Appetite.

[40] Wardle J., Steptoe A. (2003). Socioeconomic differences in attitudes and beliefs about healthy lifestyles. J. Epidemiol. Community Health.

[41] Birch L.L. (1999). Development of food preferences. Annu. Rev. Nutr..

[42] Worsley A. (2002). Nutrition knowledge and food consumption: Can nutrition knowledge change food behaviour?. Asia Pac. J. Clin. Nutr..

[43] Carson J.A., Gillham M.B., Kirk L.M., Reddy S.T., Battles J.B. (2002). Enhancing self-efficacy and patient care with cardiovascular nutrition education. Am. J. Prev. Med..

[44] Kristjansdottir A.G., Thorsdottir I., De Bourdeaudhuij I., Due P., Wind M., Klepp K.I. (2006). Determinants of fruit and vegetable intake among 11-year-old schoolchildren in a country of traditionally low fruit and vegetable consumption. Int. J. Behav. Nutr. Phys. Act..

