# Supplementary material for: Knowledge and Attitudes Are Related to Selected Salt-Specific Behaviours among Australian Parents
Source: Nutrients. 2018 Jun 4;10(6):720. doi: 10.3390/nu10060720 (PMC6024726; doi:10.3390/nu10060720)
Supplement: Supplementary file 1 [file nutrients-10-00720-s001.zip › nutrients-305111-SI/Supplementary file 1-questionnaire.docx]

**Table 1. Overview of questions included in survey**

| **Question Type** | **Questions to include** | **No. of questions** |
| --- | --- | --- |
| Demographic Questions | - Sex  - Age  - Country of birth  - Language  - Residential postcode  - Highest level of education attained  - CVD related medical conditions  - Medication for BP (if answered yes to high blood pressure)  - Dietary advice on limiting salt from health professional  - Weight  - Height  - Main shopper in household | 11 (Qs 1-11) |
| Knowledge | - Q12 Relationship between salt and sodium  - Q13 Knowledge of how much salt Australians consume  - Q14 Main food source of salt in the diet  - Q15 Salt intake recommendations  - Q17 Is salt harmful to health  - Q18 Associated health risks | 6 |
| Attitudes | - Q16 Personal attitude to own salt intake  - Q19 Concern about salt and other food related issues  - Q20 Block question. Series of agree/disagree statements to attitudes related to salt intake  - Q21 Responsibility for salt reduction | 4 |
| Behaviours | - Q22 Salt use at the table  - Q23 Salt use during cooking  - Q24 Salt shaker placed on table  - Q25 Trying to reduce salt in diet  - Q26 Block question, assessing salt related behaviours in previous month (e.g. reading labels, take-out foods, using herbs/spices) | 5 |
| Health star food label | - Q27 Question about use of health star label | 1 |
| Awareness of VicHealth salt initiative | - Q28 Awareness of VicHealth salt reduction campaign | 1 |
| Parent | - Q29 One question to determine if participant is a parent or primary carer of child/children <18 years | 1 |
| **Total Questions** |  | **29** |
| Additional Questions for parents/caretakers | - Q1 Age of child/children  - Q2 Salt use in food preparation  - Q3 Salt shaker on table  - Q4 Child’s salt use at table  - Q5 Attitude to Australian children’s salt intake  - Q6 Importance of limiting salt in their child’s diet  - Q7 Knowledge of long term health effects in children  - Q8 Should more action be taken to reduce salt in foods targeted at children | 8 |

* Note all questions have been set as forced entry. Some sensitive demographic questions, such as education level, have a ‘prefer not to answer’ response. For height and weight, there is a don’t know/prefer not to answer option for participants who are part of Facebook, consumer research panel or shopping centre survey – survey only sample. For shopping centre participants with a urine sample these questions are mandatory.

**Victorian Survey – Knowledge, Attitudes and Behaviours related to Dietary Salt Intake**

Note: Additional info below only to appear on consumer intercept survey (i.e. not online surveys via Facebook recruitment and consumer research panel)

Has the participant agreed to collect their urine?

| Yes |
| --- |
| No |

What is the participants allocated identification number?

__________

*END OF QUESTIONS TO BE COMPLETED BY STAFF MEMBER*

Thank you for taking the time to complete this survey. There are two sections to this survey. Please answer each question by selecting the most suitable option.

All responses on this survey will remain anonymous.

**SECTION ONE – DEMOGRAPHIC INFORMATION**

*In this section we are interested in finding out a little bit of background information about you. This information will help us compare the answers of respondents from different backgrounds.*

Q1. What is your sex? *Source: (*[*Australian Institute of Health and Welfare., 2012*](#_ENREF_3)*)*

| Male |
| --- |
| Female |

Q2. What is your age in complete years? *Source: (*[*Australian Institute of Health and Welfare., 2012*](#_ENREF_3)*)*

______________

Q3. In which country were you born? *Source: (*[*VicHealth, 2012*](#_ENREF_13)*).*

| Australia (includes External Territories) |
| --- |
| United Kingdom (incl. England, Scotland, Wales, Northern Ireland) |
| New Zealand |
| Italy |
| Greece |
| China |
| Vietnam |
| Lebanon |
| Other, please specify _____________________ |
| Don’t know |
| Prefer not to answer |

Q4. Do you speak a language other than English at home?

*(If more than one language, indicate the one that is spoken most often)* *Source: (*[*VicHealth, 2012*](#_ENREF_13)*).*

| No, English only |
| --- |
| Yes, Italian |
| Yes, Greek |
| Yes, Cantonese |
| Yes, Mandarin |
| Yes, Arabic |
| Yes, Vietnamese |
| Yes, German |
| Yes, Spanish |
| Yes, Tagalog (Filipino) |
| Yes, Other (please specify) ____________________ |
| Don’t know |
| Prefer not to answer |

Q5. What is the postcode of your residential address?

__________

Q6. What is the highest level of education and training you have completed? *Source: (*[*VicHealth, 2012*](#_ENREF_13)*)*

| Never attended school |
| --- |
| Some primary school |
| Completed primary school |
| Some high school (i.e Year 7 to Year 11, Form 1 to Form 5) |
| Completed high school (i.e. Year 12, Form 6, HSC) |
| TAFE or Trade Certificate or Diploma but did not complete Year 12 at secondary school |
| TAFE or Trade Certificate or Diploma and also completed Year 12 at secondary school. |
| University, or some other Tertiary Institute degree, including post university (i.e. postgraduate diploma, Master’s degree, PhD) |
| Other, please specify _______________________ |
| Don’t know |
| Prefer not to answer |

Q7. Have you ever been diagnosed with or suffered from one or more of the following conditions? *(You may select more than one)*

| Heart disease |
| --- |
| Stroke |
| High blood pressure |
| Heart attack |
| Other (please specify: __________) |
| Can’t recall/ Don’t know |
| No |

Q7.1 (if Yes to High blood pressure response)

Do you currently take medication for the control of your blood pressure? *Source: (*[*Booth, 2007*](#_ENREF_4)*)*

| Yes |
| --- |
| No |

Q8. Have you ever received any advice from your doctor or a health professional to reduce your intake of salt/sodium and/or salty foods? *Source: (*[*Booth, 2007*](#_ENREF_4)*)*

| Yes |
| --- |
| No |
| Cant’ recall |

Q9. How tall are you without shoes?

You can enter this in cm or feet and inches. **Only fill in one option.** *Source: (*[*Australian Institute of Health and Welfare., 2012*](#_ENREF_3)*)*

____________ Centimetres (e.g. 165)

OR

____________ Feet and inches (e.g. 5 feet 4 inches)

Q10. How much do you weigh without clothes and shoes?

You can enter this in kilograms OR pounds and stones. **Only fill in one option.***(Source: (*[*Australian Institute of Health and Welfare., 2012*](#_ENREF_3)*)*

_____________ Kilograms (e.g. 74)

OR

_____________ Stones and pounds (e.g. 11st 10lbs)

Q11. Are you the main person who does the grocery shopping in your household? *Source (*[*Grimes et al., 2009*](#_ENREF_8)*)*

| Yes |
| --- |
| No |
| I share the responsibility |

**SECTION TWO – YOUR VIEWS ON SALT INTAKE**

*[Questions related to knowledge]*

In this section we are interested in finding out more about your views on dietary salt.

Q12. On Australian food products information about the amount of sodium within a food product is displayed on the food label. What is the relationship between salt and sodium? *Source: Modified (*[*Australian Division of World Action on Salt and Health., 2007*](#_ENREF_2)*,* [*Grimes et al., 2009*](#_ENREF_8)*,* [*New Zealand Food Safety Authority., 2011*](#_ENREF_9)*)*

| They are exactly the same |
| --- |
| Salt contains sodium |
| Sodium contains salt |
| Don’t know |

Q13. In general, how much salt do you think Australians eat? *Source: Modelled of (*[*Papadakis et al., 2010*](#_ENREF_11)*,* [*World Health Organization., 2015*](#_ENREF_15)*)*

| Far too much |
| --- |
| Too much |
| Just the right amount |
| Too little |
| Far too little |
| Don’t know |

Q14. Which of the following do you think is the main source of salt in the Australian diet? *Source: Modelled of (*[*Australian Division of World Action on Salt and Health., 2007*](#_ENREF_2)*,* [*Papadakis et al., 2010*](#_ENREF_11)*)*

| Salt added during cooking or at the table |
| --- |
| Salt from processed foods such as breads, sausages and cheese |
| Salt from natural food sources |
| Don’t know |

Q15. Health professionals recommend that we should eat no more than a certain amount of salt **each day**. How much salt do you think this is? *Source: Modelled of (*[*Grimes et al., 2009*](#_ENREF_8)*,* [*New Zealand Food Safety Authority., 2011*](#_ENREF_9)*,* [*Sarmugam et al., 2014*](#_ENREF_12)*)*

| 3 grams (about ½ a teaspoon) |
| --- |
| 5 grams (about 1 teaspoon) |
| 8 grams (about 1 and a ½ teaspoons) |
| 10 grams (about 2 teaspoons) |
| 15 grams (about 3 teaspoons) |
| Don’t know |

Q16. How do you think your daily salt intake compares to the amount of salt recommended by health professionals? *Source: Modified (*[*Australian Division of World Action on Salt and Health., 2007*](#_ENREF_2)*,* [*Grimes et al., 2009*](#_ENREF_8)*)* [**Note Q16 is an attitude questions**]

| I eat less salt than recommended |
| --- |
| I eat about the right amount of salt |
| I eat more salt than recommended |
| I don’t know |

Q17. Do you think that eating too much salt could damage your health? *Source: (*[*Consensus Action on Salt and Health., 2009*](#_ENREF_5)*,* [*World Health Organization and Pan American Health Organization, 2010*](#_ENREF_14)*)*

| Yes |
| --- |
| No |
| Don’t know |

Q18. Which, if any, of the following conditions do you think is linked to eating too much salt?

**Please make sure you select an option for each line.** *Source: Modelled of (*[*Australian Division of World Action on Salt and Health., 2007*](#_ENREF_2)*,* [*Consensus Action on Salt and Health., 2009*](#_ENREF_5)*,* [*Grimes et al., 2009*](#_ENREF_8)*,* [*World Health Organization., 2015*](#_ENREF_15)*)*

| **Health problem** | **Yes** | **No** | **Don’t know/Not sure** |
| --- | --- | --- | --- |
| High bood pressure |  |  |  |
| Kidney disease |  |  |  |
| Heart disease/heart attack |  |  |  |
| Stroke |  |  |  |
| Stomach cancer |  |  |  |

*[Questions related to attitudes]*

Q19. Please indicate on the scale below how concerned you are about each of the following food related issues?

**Please make sure you select an option for each line.** *Source: Modelled of (*[*Arcand et al., 2013*](#_ENREF_1)*,* [*Food Standards Agency and COI Communications., 2005*](#_ENREF_6)*)*

|  | Not at all concerned | Not very concerned | Somewhat concerned | Very concerned | Extremely concerned |
| --- | --- | --- | --- | --- | --- |
| Healthy eating |  |  |  |  |  |
| The amount of sugar in food |  |  |  |  |  |
| The amount of salt in food |  |  |  |  |  |
| The amount of fat in food |  |  |  |  |  |
| The amount of saturated fat in food |  |  |  |  |  |
| The amount of kilojoules/calories in food |  |  |  |  |  |

Q20. Please indicate on the scale below how much you agree or disagree with the following statements.

**Please make sure you select an option for each line.** *Source: Modified (*[*Arcand et al., 2013*](#_ENREF_1)*,* [*Grimes et al., 2009*](#_ENREF_8)*)*

| **Statement** | **Strongly disagree** | **Disagree** | **Neither agree nor disagree** | **Agree** | **Strongly agree** |
| --- | --- | --- | --- | --- | --- |
| Himalayan salt, pink salt, sea salt and gourmet salts are healthier than regular table salt |  |  |  |  |  |
| I believe salt needs to be added to food to make it tasty |  |  |  |  |  |
| My health would improve if I reduced the amount of salt in my diet |  |  |  |  |  |
| It is hard to understand sodium information displayed on food labels |  |  |  |  |  |
| When eating out at restaurants/cafes/pubs, I find that lower salt options are not readily available or only in limited variety |  |  |  |  |  |
| There should be laws which limit the amount of salt added to manufactured foods |  |  |  |  |  |

Q21. From the list below please rate how responsible you think each group is for reducing the amount of salt Australians eat.

**Please make sure you select an option for each line.**  *Source: Modified (*[*Newson et al., 2013*](#_ENREF_10)*)*

| **Group** | **Not at all responsible** | **Somewhat responsible** | **Responsible** | **Very responsible** | **Don’t know** |
| --- | --- | --- | --- | --- | --- |
| Government |  |  |  |  |  |
| Food manufacturers |  |  |  |  |  |
| Business (e.g. supermarkets, local markets) |  |  |  |  |  |
| Chefs preparing foods in restuarants/pubs/cafes |  |  |  |  |  |
| Friends/family |  |  |  |  |  |
| Yourself |  |  |  |  |  |
| Fast food chains |  |  |  |  |  |

*[Questions related to behaviours]*

Q22. How often do you add salt to your food at the table? *Source: (*[*World Health Organization., 2015*](#_ENREF_15)*)*

| Always |
| --- |
| Often |
| Sometimes |
| Rarely |
| Never |
| Don’t know |

Q23. In the food you eat at home, how often is salt added during cooking? *Source: (*[*World Health Organization., 2015*](#_ENREF_15)*)*

| Always |
| --- |
| Often |
| Sometimes |
| Rarely |
| Never |
| Don’t know |

Q24. Do you place a salt shaker on your table at meal times? *Source: Modified (*[*Grimes et al., 2015*](#_ENREF_7)*)*

| Always |
| --- |
| Often |
| Sometimes |
| Rarely |
| Never |
| Don’t know |

Q25. Are you trying to cut down on the amount of salt you eat? *Source: Modified (*[*Food Standards Agency and COI Communications., 2005*](#_ENREF_6)*,* [*New Zealand Food Safety Authority., 2011*](#_ENREF_9)*,* [*World Health Organization and Pan American Health Organization, 2010*](#_ENREF_14)*,* [*World Health Organization., 2015*](#_ENREF_15)*)*

| Yes |
| --- |
| No |
| Don’t know |

Q26. Below are some common ways to lower the amount of salt in your diet. In the past month, to what extent have you personally done any of the following?

**Please make sure you select an option for each line.** *Source modelled from:(*[*Arcand et al., 2013*](#_ENREF_1)*,* [*World Health Organization., 2015*](#_ENREF_15)*)*

|  | Never do this | Rarely do this | Sometimes do this | Often do this | Always do this | Does not apply to me |
| --- | --- | --- | --- | --- | --- | --- |
| Looked at a food label to check the salt/sodium content of a food item |  |  |  |  |  |  |
| Avoided eating packaged, ready-to-eat foods |  |  |  |  |  |  |
| Used spices/herbs instead of salt when cooking |  |  |  |  |  |  |
| Avoided eating food from fast food restaurants (e.g. McDonalds, KFC, Pizza Hut) |  |  |  |  |  |  |
| Avoided eating food from an Asian style restaurant or takeaway store (e.g. Chinese, Thai, Indian) |  |  |  |  |  |  |
| Purchased foods labelled “no added salt”, “salt reduced” or “reduced sodium” |  |  |  |  |  |  |
| When eating out, asked to have your meal prepared without salt |  |  |  |  |  |  |

Q27. On some food products a health star rating is displayed on the food label.

*[insert image of health star rating label]*

Do you use the health star rating information to choose any of the following products?

|  | Usually | Sometimes | Never | Don’t know |
| --- | --- | --- | --- | --- |
| Breakfast cereals |  |  |  |  |
| Cheese |  |  |  |  |
| Bread |  |  |  |  |

Q28. Are you aware of VicHealth’s initiative to reduce salt intake within the Victorian population?

| Yes |
| --- |
| No |
| Don’t know |

Q29. Are you a parent or caregiver for a child or children under the age of 18 years?

| No |
| --- |
| Yes, I’m a parent |
| Yes, I care for a child/children |

*(if no directed to end of survey)*

**ADDITIONAL QUESTIONS FOR PARENTS**

You have indicated that you are a parent or carer of a child or children. Because we are interested in what parents think about salt in children's diets we would like to ask you 8 more questions.  This will take about **5 minutes**to complete**.**We greatly appreciate your time.

Q1. How old is your child/children or the child/children that you care for?

**You can select more than one age category if needed.**

| 0-1 year |
| --- |
| 2-4 years |
| 5-12 years |
| 13-17 years |

Q2. Do you add salt to foods you prepare for your child or children? *Source modified: (*[*World Health Organization., 2015*](#_ENREF_15)*)*

| Always |
| --- |
| Often |
| Sometimes |
| Rarely |
| Never |
| Not applicable, I do not prepare food for my child |

Q3. Do you place a salt shaker on your table at meal times? *Source: Modified (*[*Grimes et al., 2015*](#_ENREF_7)*)*

| Always |
| --- |
| Often |
| Sometimes |
| Rarely |
| Never |
| Don’t know |

Q4. Does your child or children add salt to their food at the table? *Source: Modified: (*[*World Health Organization., 2015*](#_ENREF_15)*)*

| Always |
| --- |
| Often |
| Sometimes |
| Rarely |
| Never |
| Don’t know |

Q5. In general how much salt do you think Australian children eat? *(Source: Modified to be child specific (*[*Papadakis et al., 2010*](#_ENREF_11)*,* [*World Health Organization., 2015*](#_ENREF_15)*)*

| Far too much |
| --- |
| Too much |
| Just the right amount |
| Too little |
| Far too little |
| Don’t know |

Q6. Please indicate on the scale below how much you agree or disagree with the following statement:

“Limiting the amount of salt my child/or children eat/s is important to me.”

| Strongly disagree |
| --- |
| Disagree |
| Neither agree nor disagree |
| Agree |
| Strongly agree |

Q7. Please indicate on the scale below how much you agree or disagree with the following statement:

“In the long term eating too much salt during childhood may have harmful effects on children’s health.” *Source: Modified (*[*Australian Division of World Action on Salt and Health., 2007*](#_ENREF_2)*)*

| Strongly disagree |
| --- |
| Disagree |
| Neither agree nor disagree |
| Agree |
| Strongly agree |

Q8. Medical research suggests that most Australian children eat more salt than is good for them. Do you think more action needs to be taken to reduce the salt in foods targeted at children? *Source modified: (*[*Australian Division of World Action on Salt and Health., 2007*](#_ENREF_2)*)*

| Strongly disagree |
| --- |
| Disagree |
| Neither agree nor disagree |
| Agree |
| Strongly agree |

You have completed the Survey! Please hit the submit button.

Thank you for your time.

**References**

ARCAND, J., MENDOZA, J., QI, Y., HENSON, S., LOU, W. & L'ABBE, M. R. 2013. Results of a National Survey Examining Canadians' Concern, Actions, Barriers, and Support for Dietary Sodium Reduction Interventions. *Canadian Journal of Cardiology,* 29**,** 628-631.

AUSTRALIAN DIVISION OF WORLD ACTION ON SALT AND HEALTH. 2007. *2007 Survey of Australian Consumer Awareness and Practices Relating to Salt Report* [Online]. Available: <http://www.awash.org.au/wp-content/uploads/2012/10/AWASH_ConsumerSurveyReport_2007_05_15.pdf> [Accessed 26th May 2015.

AUSTRALIAN INSTITUTE OF HEALTH AND WELFARE. 2012. National Health Data Dictionary version 16. Canberra.

BOOTH, A. 2007. *Implementation strategies and effectiveness of nutritional messages for improving health.* Ph.D., Deakin University.

CONSENSUS ACTION ON SALT AND HEALTH. 2009. *Salt and Your Health. TNS Public Opinion Survey* [Online]. Available: <http://www.actiononsalt.org.uk/Docs/33386.pdf> [Accessed 5th Aug 2015.

FOOD STANDARDS AGENCY & COI COMMUNICATIONS. 2005. Consumer Attitudes to Food Standards Wave 5 - 2004. London.

GRIMES, C. A., BAXTER, J. R., CAMPBELL, K. J., RIDDELL, L. J., RIGO, M., LIEM, D. G., KEAST, R. S., HE, F. J. & NOWSON, C. A. 2015. Cross-Sectional Study of 24-Hour Urinary Electrolyte Excretion and Associated Health Outcomes in a Convenience Sample of Australian Primary Schoolchildren: The Salt and Other Nutrients in Children (SONIC) Study Protocol. *JMIR Res Protoc,* 4**,** e7.

GRIMES, C. A., RIDDELL, L. J. & NOWSON, C. A. 2009. Consumer knowledge and attitudes to salt intake and labelled salt information. *Appetite,* 53**,** 189-94.

NEW ZEALAND FOOD SAFETY AUTHORITY. 2011. *Salt Consumer Survey* [Online]. Available: <http://www.foodsafety.govt.nz/elibrary/industry/salt-survey.pdf> [Accessed 26th May 2015.

NEWSON, R. S., ELMADFA, I., BIRO, G., CHENG, Y., PRAKASH, V., RUST, P., BARNA, M., LION, R., MEIJER, G. W., NEUFINGERL, N., SZABOLCS, I., VAN ZWEDEN, R., YANG, Y. & FEUNEKES, G. I. 2013. Barriers for progress in salt reduction in the general population. An international study. *Appetite,* 71**,** 22-31.

PAPADAKIS, S., PIPE, A. L., MOROZ, I. A., REID, R. D., BLANCHARD, C. M., COTE, D. F. & MARK, A. E. 2010. Knowledge, attitudes and behaviours related to dietary sodium among 35- to 50-year-old Ontario residents. *Can J Cardiol,* 26**,** e164-9.

SARMUGAM, R., WORSLEY, A. & FLOOD, V. 2014. Development and validation of a salt knowledge questionnaire. *Public Health Nutr,* 17**,** 1061-8.

VICHEALTH. 2012. *VicHealth Indicators Survey 2011* [Online]. Available: https://[www.vichealth.vic.gov.au/programs-and-projects/vichealth-indicators-survey-2011](http://www.vichealth.vic.gov.au/programs-and-projects/vichealth-indicators-survey-2011) [Accessed 23 Sep 2016.

WORLD HEALTH ORGANIZATION & PAN AMERICAN HEALTH ORGANIZATION 2010. Protocol for Population Level Sodium Determination in 24-hr Urine Samples. Canada: WHO/PAHO Regional Expert Group for Cardiovascular Disease Prevention through Population-wide Dietary Salt Reduction.

WORLD HEALTH ORGANIZATION. 2015. *WHO STEPS Instrument (Core and Expanded) v3.1* [Online]. Available: <http://www.who.int/chp/steps/instrument/en/> [Accessed 26th May 2015.
