# Supplementary material for: Knowledge and Attitudes Are Related to Selected Salt-Specific Behaviours among Australian Parents
Source: Nutrients. 2018 Jun 4;10(6):720. doi: 10.3390/nu10060720 (PMC6024726; doi:10.3390/nu10060720)
Supplement: Supplementary file 1 [file nutrients-10-00720-s001.zip › nutrients-305111-SI/Table S1.docx]

**Table S1.** Proportion of parents who reported engaging in specific behaviours to reduce salt in their diet over the past month (n=837)

| **Behaviour*** | **N (%) Reporting** |
| --- | --- |
| **Looked at a food label to check the salt/sodium content of a food item** | |
| Always/often | 259 (31%) |
| Sometimes | 228 (27%) |
| Rarely/never | 338 (40%) |
| **Avoided eating packaged, ready-to-eat foods** | |
| Always/often | 381 (46%) |
| Sometimes | 252 (30%) |
| Rarely/never | 190 (23%) |
| **Used spices/herbs instead of salt when cooking** | |
| Always/often | 445 (53%) |
| Sometimes | 212 (25%) |
| Rarely/never | 154 (18%) |
| **Avoided eating food from fast food restaurants (e.g. McDonalds, KFC, Pizza Hut)** | |
| Always/often | 422 (50%) |
| Sometimes | 225 (27%) |
| Rarely/never | 176 (21%) |
| **Avoided eating from an Asian style restaurant or takeaway store (i.e. Chinese, Thai, Indian)** | |
| Always/often | 274 (33%) |
| Sometimes | 263 (31%) |
| Rarely/never | 272 (33%) |
| **Purchased foods labelled ‘no added salt’, ‘salt reduced’ or ‘reduced sodium’** | |
| Always/often | 326 (39%) |
| Sometimes | 252 (30%) |
| Rarely/never | 243 (29%) |
| **When eating out, asked to have your meal prepared without salt** | |
| Always/often | 134 (16%) |
| Sometimes | 107 (13%) |
| Rarely/never | 578 (69%) |

^*^Behaviour percentages may not add to 100% as those who responded ‘does not apply to me’ were excluded
