# Supplementary material for: Knowledge and Attitudes Are Related to Selected Salt-Specific Behaviours among Australian Parents
Source: Nutrients. 2018 Jun 4;10(6):720. doi: 10.3390/nu10060720 (PMC6024726; doi:10.3390/nu10060720)
Supplement: Supplementary file 1 [file nutrients-10-00720-s001.zip › nutrients-305111-SI/Table S2.docx]

**Table S2.** Association of parents’ knowledge related to salt intake in adults and salt-related behaviours

| ***Knowledge*** | ***Behaviours*** | | | ***Knowledge*** | ***Behaviours*** | | |
| --- | --- | --- | --- | --- | --- | --- | --- |
|  | *Adding salt at the table* | | |  | *Adding salt at the table* | | |
| Eating too much salt could damage your health | **Always/**  **Often***  **(%)** | **Rarely/**  **Never**  **(%)** | **p-value**  **(χ^2^)** | Australians eat too much salt | **Always/**  **Often***  **(%)** | **Rarely/**  **Never**  **(%)** | **p-value**  **(χ^2^)** |
| **Yes (%)** | 52 | 48 | **0.001** | **Far too much/Too much (%)** | 51 | 49 | **<0.001** |
| **No/Don’t know (%)** | 72 | 28 |  | **Too little/Far too little/don’t know (%)** | 69 | 31 |  |
|  | *Adding salt during cooking* | | |  | *Adding salt during cooking* | | |
|  | **Always/**  **Often**  **(%)** | **Rarely/**  **Never**  **(%)** |  |  | **Always/**  **Often**  **(%)** | **Rarely/**  **Never**  **(%)** |  |
| **Yes (%)** | 66 | 34 | **0.004** | **Far too much/Too much (%)** | 44 | 56 | **<0.001** |
| **No/Don’t know (%)** | 82 | 18 |  | **Too little/Far too little/don’t know (%)** | 59 | 41 |  |
|  | *Placing salt shaker on table at meal times* | | |  | *Placing salt shaker on table at meal times* | | |
|  | **Always/**  **Often**  **(%)** | **Rarely/**  **Never**  **(%)** |  |  | **Always/**  **Often**  **(%)** | **Rarely/**  **Never**  **(%)** |  |
| **Yes (%)** | 49 | 51 | **0.011** | **Far too much/Too much (%)** | 48 | 52 | **0.038** |
| **No/Don’t know (%)** | 64 | 36 |  | **Too little/Far too little/don’t know (%)** | 58 | 42 |  |
|  | *Avoided eating food from fast food restaurants* | | |  | *Avoided eating food from fast food restaurants* | | |
|  | **Always/**  **Often**  **(%)** | **Rarely/**  **Never**  **(%)** |  |  | **Always/**  **Often**  **(%)** | **Rarely/**  **Never**  **(%)** |  |
| **Yes (%)** | 80 | 20 | **0.005** | **Far too much/Too much (%)** | 79 | 21 | 0.408 |
| **No/Don’t know (%)** | 65 | 35 |  | **Too little/Far too little/don’t know (%)** | 76 | 24 |  |
|  | *Avoided eating packaged, ready-to-eat foods* | | |  | *Avoided eating packaged, ready-to-eat foods* | | |
|  | **Always/**  **Often**  **(%)** | **Rarely/**  **Never**  **(%)** |  |  | **Always/**  **Often**  **(%)** | **Rarely/**  **Never**  **(%)** |  |
| **Yes (%)** | 79 | 21 | **<0.001** | **Far too much/Too much (%)** | 77 | 23 | 0.539 |
| **No/Don’t know (%)** | 56 | 44 |  | **Too little/Far too little/don’t know (%)** | 75 | 25 |  |
|  | *Purchased foods labelled “no added salt”, “salt reduced” or “reduced sodium* | | |  | *Purchased foods labelled “no added salt”, “salt reduced” or “reduced sodium* | | |
|  | **Always/**  **Often**  **(%)** | **Rarely/**  **Never**  **(%)** |  |  | **Always/**  **Often**  **(%)** | **Rarely/**  **Never**  **(%)** |  |
| **Yes (%)** | 73 | 27 | **<0.001** | **Far too much/Too much (%)** | 72 | 28 | **0.006** |
| **No/Don’t know (%)** | 47 | 53 |  | **Too little/Far too little/don’t know (%)** | 60 | 40 |  |
|  |  |  |  |  |  | |  |
|  | *When eating out, asked to have your meal prepared without salt* | | |  | *When eating out, asked to have your meal prepared without salt* | | |
|  | **Always/**  **Often**  **(%)** | **Rarely/**  **Never**  **(%)** |  |  | **Always/**  **Often**  **(%)** | **Rarely/**  **Never**  **(%)** |  |
| **Yes (%)** | 29 | 71 | 0.510 | **Far too much/Too much (%)** | 27 | 73 | **<0.001** |
| **No/Don’t know (%)** | 33 | 67 |  | **Too little/Far too little/don’t know (%)** | 45 | 55 |  |
|  | *Look at a food label to check the salt/sodium content of a food* | | |  | *Look at a food label to check the salt/sodium content of a food* | | |
|  | **Always/**  **Often**  **(%)** | **Rarely/**  **Never**  **(%)** |  |  | **Always/**  **Often**  **(%)** | **Rarely/**  **Never**  **(%)** |  |
| **Yes (%)** | 61 | 39 | **<0.001** | **Far too much/Too much (%)** | 59 | 41 | 0.633 |
| **No/Don’t know (%)** | 35 | 65 |  | **Too little/Far too little/don’t know (%)** | 61 | 39 |  |
|  | *Avoided eating food from an Asian style restaurant or takeaway store* | | |  | *Avoided eating food from an Asian style restaurant or takeaway store* | | |
|  | **Always/**  **Often**  **(%)** | **Rarely/**  **Never**  **(%)** |  |  | **Always/**  **Often**  **(%)** | **Rarely/**  **Never**  **(%)** |  |
| **Yes (%)** | 68 | 32 | **0.003** | **Far too much/Too much (%)** | 66 | 34 | 0.628 |
| **No/Don’t know (%)** | 53 | 47 |  | **Too little/Far too little/don’t know (%)** | 68 | 32 |  |
|  | *Used spices/herbs instead of salt when cooking* | | |  | *Used spices/herbs instead of salt when cooking* | | |
|  | **Always/**  **Often**  **(%)** | **Rarely/**  **Never**  **(%)** |  |  | **Always/**  **Often**  **(%)** | **Rarely/**  **Never**  **(%)** |  |
| **Yes (%)** | 82 | 18 | **0.002** | **Far too much/Too much (%)** | 81 | 19 | 0.575 |
| **No/Don’t know (%)** | 67 | 33 |  | **Too little/Far too little/don’t know (%)** | 79 | 21 |  |

*Response also includes ‘Sometimes’
