# Supplementary material for: Knowledge and Attitudes Are Related to Selected Salt-Specific Behaviours among Australian Parents
Source: Nutrients. 2018 Jun 4;10(6):720. doi: 10.3390/nu10060720 (PMC6024726; doi:10.3390/nu10060720)
Supplement: Supplementary file 1 [file nutrients-10-00720-s001.zip › nutrients-305111-SI/Table S3.docx]

**Table S3.** Association of parents’ knowledge and attitudes related to their child/children with specific salt-related behaviours

| ***Knowledge*** | ***Behaviours*** | | | ***Knowledge*** | ***Behaviours*** | | | ***Attitude*** | ***Behaviours*** | |  |  |
| --- | --- | --- | --- | --- | --- | --- | --- | --- | --- | --- | --- | --- |
|  | *Avoided eating food from fast food restaurants* | | |  | *Avoided eating food from fast food restaurants* | | |  | *Avoided eating food from fast food restaurants* | |  | |
| In the long-term, eating too much salt can damage children’s health | **Always/**  **Often***  **(%)** | **Rarely/**  **Never**  **(%)** | **P-value**  **(χ^2^)** | Children eat far too much/too much salt | **Always/**  **Often**  **(%)** | **Rarely/**  **Never**  **(%)** | **P-value**  **(χ^2^)** | Limiting the amount of salt my child eats is important to me | **Always/**  **Often**  **(%)** | **Rarely/**  **Never**  **(%)** | **P-value**  **(χ^2^)** | |
| **Strongly agree/**  **Agree (%)** | 81 | 19 | **0.005** | **Yes (%)** | 79 | 21 | 0.647 | **Strongly agree/**  **Agree (%)** | 82 | 18 | **<0.001** | |
| **Strongly disagree/**  **disagree** (%)** | 71 | 29 |  | **No (%)** | 78 | 22 |  | **Strongly disagree/**  **Disagree (%)** | 70 | 30 |  |  |
|  | *Avoided eating packaged, ready-to-eat foods* | | |  | *Avoided eating packaged, ready-to-eat foods* | | |  | *Avoided eating packaged, ready-to-eat foods* | |  | |
|  | **Always/**  **Often**  **(%)** | **Rarely/**  **Never**  **(%)** |  |  | **Always/**  **Often**  **(%)** | **Rarely/**  **Never**  **(%)** |  |  | **Always/**  **Often**  **(%)** | **Rarely/**  **Never**  **(%)** |  | |
| **Strongly agree/**  **Agree (%)** | 80 | 20 | **<0.001** | **Yes (%)** | 78 | 22 | 0.357 | **Strongly agree/**  **Agree (%)** | 81 | 19 | **<0.001** | |
| **Strongly disagree/**  **Disagree (%)** | 66 | 34 |  | **No (%)** | 75 | 25 |  | **Strongly disagree/**  **Disagree (%)** | 66 | 34 |  |  |
|  | *Purchased foods labelled “no added salt”, “salt reduced” or “reduced sodium* | | |  | *Purchased foods labelled “no added salt”, “salt reduced” or “reduced sodium* | | |  | *Purchased foods labelled “no added salt”, “salt reduced” or “reduced sodium* | | | |
|  | **Always/**  **Often**  **(%)** | **Rarely/**  **Never**  **(%)** |  |  | **Always/**  **Often**  **(%)** | **Rarely/**  **Never**  **(%)** |  |  | **Always/**  **Often**  **(%)** | **Rarely/**  **Never**  **(%)** |  | |
| **Strongly agree/**  **Agree (%)** | 75 | 25 | **<0.001** | **Yes (%)** | 73 | 27 | **0.010** | **Strongly agree/**  **Agree (%)** | 77 | 23 | **<0.001** | |
| **Strongly disagree/**  **Disagree (%)** | 56 | 44 |  | **No (%)** | 64 | 36 |  | **Strongly disagree/**  **Disagree (%)** | 55 | 45 |  |  |
|  | *When eating out, asked to have your meal prepared without salt* | | |  | *When eating out, asked to have your meal prepared without salt* | | |  | *When eating out, asked to have your meal prepared without salt* | | | |
|  | **Always/**  **Often**  **(%)** | **Rarely/**  **Never**  **(%)** |  |  | **Always/**  **Often**  **(%)** | **Rarely/**  **Never**  **(%)** |  |  | **Always/**  **Often**  **(%)** | **Rarely/**  **Never**  **(%)** |  | |
| **Strongly agree/**  **Agree (%)** | 27 | 73 | **0.001** | **Yes (%)** | 24 | 76 | **<0.001** | **Strongly agree/**  **Agree (%)** | 28 | 72 | 0.158 | |
| **Strongly disagree/**  **Disagree (%)** | 39 | 61 |  | **No (%)** | 44 | 56 |  | **Strongly disagree/**  **Disagree (%)** | 33 | 67 |  |  |
|  | *Looked at a food label to check the salt/sodium content of a food* | | |  | *Looked at a food label to check the salt/sodium content of a food* | | |  | *Looked at a food label to check the salt/sodium content of a food* | | | |
|  | **Always/**  **Often**  **(%)** | **Rarely/**  **Never**  **(%)** |  |  | **Always/**  **Often**  **(%)** | **Rarely/**  **Never**  **(%)** |  |  | **Always/**  **Often**  **(%)** | **Rarely/**  **Never**  **(%)** |  | |
| **Strongly agree/**  **Agree (%)** | 62 | 38 | **0.001** | **Yes (%)** | 60 | 40 | 0.314 | **Strongly agree/**  **Agree (%)** | 64 | 36 | **<0.001** | |
| **Strongly disagree/**  **Disagree (%)** | 49 | 51 |  | **No (%)** | 56 | 44 |  | **Strongly disagree/**  **Disagree (%)** | 48 | 52 |  |  |
|  | *Avoided eating food from an Asian style restaurant or takeaway store* | | |  | *Avoided eating food from an Asian style restaurant or takeaway store* | | |  | *Avoided eating food from an Asian style restaurant or takeaway store* | | | |
|  | **Always/**  **Often**  **(%)** | **Rarely/**  **Never**  **(%)** |  |  | **Always/**  **Often**  **(%)** | **Rarely/**  **Never**  **(%)** |  |  | **Always/**  **Often**  **(%)** | **Rarely/**  **Never**  **(%)** |  | |
| **Strongly agree/**  **Agree (%)** | 68 | 32 | **0.047** | **Yes (%)** | 66 | 34 | 0.873 | **Strongly agree/**  **Agree (%)** | 71 | 29 | **<0.001** | |
| **Strongly disagree/**  **Disagree (%)** | 60 | 40 |  | **No (%)** | 67 | 33 |  | **Strongly disagree/**  **Disagree (%)** | 56 | 44 |  |  |
|  | *Used spices/herbs instead of salt when cooking* | | |  | *Used spices/herbs instead of salt when cooking* | | |  | *Used spices/herbs instead of salt when cooking* | | | |
|  | **Always/**  **Often**  **(%)** | **Rarely/**  **Never**  **(%)** |  |  | **Always/**  **Often**  **(%)** | **Rarely/**  **Never**  **(%)** |  |  | **Always/**  **Often**  **(%)** | **Rarely/**  **Never**  **(%)** |  | |
| **Strongly agree/**  **Agree (%)** | 83 | 17 | **0.012** | **Yes (%)** | 82 | 18 | 0.226 | **Strongly agree/**  **Agree (%)** | 84 | 16 | **0.004** | |
| **Strongly disagree/**  **Disagree (%)** | 75 | 25 |  | **No (%)** | 78 | 22 |  | **Strongly disagree/**  **Disagree (%)** | 75 | 25 |  |  |

*Response also includes ‘Sometimes’

**Response also includes ‘Neither agree nor disagree’
